# Identifying Trusted Sources of Lyme Disease Prevention Information Among Internet Users Connected to Academic Public Health Resources: Internet-Based Survey Study

**DOI:** 10.2196/43516

**Published:** 2023-07-26

**Authors:** Heather L Kopsco, Rayda K Krell, Thomas N Mather, Neeta P Connally

**Affiliations:** 1 Department of Pathobiology College of Veterinary Medicine University of Illinois Urbana-Champaign Urbana, IL United States; 2 Department of Biological and Environmental Sciences Western Connecticut State University Danbury, CT United States; 3 Department of Plant Sciences and Entomology University of Rhode Island Kingston, RI United States

**Keywords:** communication, consumer health information, disease, internet, Lyme disease, online, pathogen, prevention, public health, resources, social media, survey, tickborne disease, ticks

## Abstract

**Background:**

Misinformation about Lyme disease and other tick-transmitted pathogens circulates frequently on the internet and can compete with, or even overshadow, science-based guidance on tick-borne disease (TBD) prevention.

**Objective:**

We surveyed internet users connected to academic tick-related resources to identify trusted sources of Lyme disease prevention information, explore confidence in tick bite prevention information, and examine associations of these responses with answers to commonly disputed issues.

**Methods:**

The survey was conducted through social media and website pages for Western Connecticut State University Tickborne Disease Prevention Laboratory and the University of Rhode Island TickEncounter Resource Center.

**Results:**

Respondents (N=1190) were predominantly female (903/1190, 76.3%), middle-aged (574/1182, 48.6%), and resided in New England states (663/1190, 55.7%). In total 984 of 1186 (83%) respondents identified conventional experts (eg, the Centers for Disease Control [CDC] or other government health agencies, physicians who follow Infectious Diseases Society of America guidelines for Lyme disease treatment guidelines, and academics) as trustworthy TBD prevention resources. However, nearly one-fourth of respondents would first consult personal contacts and web-based communities regarding prevention information before consulting conventional expert sources. The opinions of public health experts and physicians were rated among the top motivators underlying personal prevention decisions; yet, more than 50% of participants revealed distrustful attitudes toward, or were uncertain about, CDC-supported statements related to time to transmission of Lyme disease (708/1190, 59.5%), the safety of diethyltoluamide-based repellents for children (604/1183, 51.1%), and recommended use of antibiotic prophylaxis (773/1181, 65.4%). Multimodal regression models revealed that participants from high-Lyme-disease-incidence states were more likely to first seek TBD prevention information from personal networks and nontraditional sources before approaching conventional sources of TBD prevention information. We found that those reporting high rates of social media usage were more than twice as likely to first seek traditional expert sources of prevention information but were overall more likely to reject CDC-promoted Lyme disease information, in particular the established time to transmission of Lyme disease bacteria. Models also predicted that those participants who disagreed with the conventional scientific view on the antibiotic prophylaxis prevention statement were less likely to be confident in their ability to protect themselves from a tick bite. Overall, uncertainty in one’s ability to protect oneself against tick bites was strongly associated with uncertainty about beliefs in CDC-promoted TBD prevention information. Self-reported trust in experts and frequency of social media use suggest that these platforms may provide opportunities to engage directly with the public about TBD prevention practices.

**Conclusions:**

Using strategies to improve public trust and provide information where the public engages on social media may improve prevention communication and adoption of best practices.

## Introduction

General public distrust of the scientific community [1,2] creates challenges for dispelling controversies with scientific evidence. Distrust of science-based disease and prevention information appears to be associated with public reliance on health information acquired from nonexpert internet and social media sources (ie, user-generated content created and exchanged on internet-based apps such as Facebook, Twitter, Reddit, and Instagram) [3-6]. The construction of narrative surrounding health and prevention information into “pseudoknowledge” is well documented [7], and studies have found numerous examples of disease and prevention information being misrepresented on internet-based platforms [8]. In one study, researchers examined Twitter data and found that users who encountered posts containing negative opinions regarding the human papillomavirus vaccine were over 3 times more likely to share information espousing these views than those who were exposed to neutral or positive information about the vaccine [9]. In another study, researchers found that Facebook users shared posts containing content asserting that Zika virus was a conspiracy theory more often than factual prevention messages, despite being provided a greater number of factual articles regarding the virus’ origins [10]. More recently, social media and internet platforms have been strongly implicated in the spread of misinformation and conspiracy theories regarding the COVID-19 pandemic and its origin, transmission, treatment, and prevention [8].

Misinformation about the tick-transmitted pathogen that causes Lyme disease similarly thrives on social media platforms [11,12]. The dissemination of information that counters scientific evidence about Lyme disease prevention presents a public health risk. Lyme disease is the most prevalent vector-borne disease in the United States [13] and accounted for 82% of all reported human tick-borne disease (TBD) cases from 2004 to 2016 [14]. Despite the disease’s ubiquity and well-studied epidemiology, public controversy regarding how it is transmitted and how best to prevent tick bites is prevalent [12,15,16]. There is continuous debate surrounding how long it takes a blacklegged tick (a.k.a. deer tick, *Ixodes scapularis*) or western blacklegged tick (*Ixodes pacificus*) to transmit an infectious dose of Lyme disease bacteria (*Borrelia burgdorferi*) [16]. There is also widespread public distrust regarding the safety of established repellents such as diethyltoluamide (DEET) and permethrin [17-19], and confusion about when and for how long a person should be treated with antibiotics after a tick bite regardless of whether the tick was known to be infected with Lyme-causing bacteria [16,20]. These disputed issues continue to fuel ideological encampments between scientists and the public when it comes to discussing Lyme disease prevention.

Although conjectural health information is common on social media and other websites, social media can also be leveraged to promote science-based health information, and public health agencies are making attempts to engage the public with scientifically based information at the interface where they seek out information by using internet and social media platforms [21]. During an Ebola outbreak, the Nigerian government responded directly on Twitter to dangerously misleading treatment and prevention information for Ebola cases that circulated on the platform, and some corrections were found still spreading among local users three days later [22]. The Nigerian experience provides an example that as much as incorrect information is spread on social media, it is also possible to leverage these platforms to spread scientifically backed information to a wide audience. However, the fact that scientists are involved in disseminating science-based public health messages may contribute to what turns off the public to the information and alter their Lyme disease prevention information searches and acceptance. In 2 recent studies examining the Lyme disease content on YouTube, researchers found that personal experience-based stories and celebrity-based videos are more frequently viewed by the public than the information from public health agencies [11]. Further, those seeking Lyme disease video content tend to express a negative perception of science-based Lyme disease prevention information [12,23], purportedly because this is a group that has had poor experiences with physicians or public health experts related to a TBD diagnosis and is seeking community that understands their struggles with illness.

Knowledge, attitudes, and beliefs regarding Lyme disease [23,24] and web-based health information–seeking behaviors [25-28] have been studied in general populations. However, it is yet unclear what sources of Lyme disease prevention information are trusted and sought by internet and social media users [28], and those who are already well connected to academic sources of TBD prevention information. We conducted a survey through 2 university-based social media and website channels to identify trusted sources of Lyme disease information among internet users who are followers of these sites. We sought to understand whether people with connections to academic sources of TBD prevention information trust sources of TBD prevention information that are consistent with public health recommendations per government agencies like the Centers for Disease Control and Prevention (CDC). Our investigation focused on identifying information in four main areas: (1) who people trust among specific, predefined traditional expert and other sources of prevention information, (2) where they first seek prevention information, (3) how these sources of trust relate to confidence in personal TBD prevention ability, and (4) how reported trust and confidence are associated with views on the disputed topics. We predicted that those who reported greater trust in established traditional expert public health sources (eg, the CDC and university scientists) would also be more likely to accept CDC-based recommendations for TBD prevention and report greater confidence in the ability to prevent a tick bite.

## Methods

### Recruitment

We conducted an internet-based Lyme disease prevention survey by posting a unique survey web link to public social media accounts (Facebook) and website associated with the University of Rhode Island’s TickEncounter Resource Center (TERC), and another unique survey link to public social media accounts (Facebook and Twitter) associated with Western Connecticut State University’s Tickborne Disease Prevention Laboratory (TDPL). The survey was created using Survey Monkey [29] and posted a single time on each platform by TERC or TDPL in October 2017. The survey link was available to internet users until January 2019 and reposted on social media sites periodically during this time. Given that the total number of followers (ie, “population”) for all social media platforms exceeded 1500 people, we sought a sample size of 300 (assuming a 95% CI and a 5% margin of error).

### Survey Tool

The 17-question survey (Multimedia Appendix 1) asked respondents to rate how likely they were to trust Lyme disease prevention information from the following sources: personal physician, close friend or family, online community forum, mainstream internet health sites (eg, WebMD), government public health agency (eg, CDC), physicians considered to be “Lyme literate,” (ie, a term for physicians who treat Lyme disease cases almost exclusively and often in accordance with the International Lyme and Associated Diseases Society [ILADS]), and pest control operator. While we recognize that there may have been variations in interpretation among survey respondents, we included the term Lyme literate due to the familiarity of this concept within the study group, rather than identifying the treatment guidelines typically followed by these practitioners. We grouped physicians who treat according to Infectious Disease Society of America (IDSA) and CDC guidelines, mainstream internet health sites, university scientists, and government public health agencies into the conventional scientific information category, while Lyme-literate physicians, close friends and family, web-based community forums, and pest control operators were placed in the unconventional information and personal network information category. The survey also asked respondents to identify the source they accessed first when seeking Lyme disease prevention information during the past year. Respondents rated their own knowledge about Lyme disease prevention as well as their confidence in protecting themselves and their family from tick bites. Survey respondents ranked their top 3 decision-making factors in making tick bite prevention decisions among 7 potential influences including the opinions of public health experts or primary care physicians, concern for the environment, product cost, friends and family opinion, and personal intuition. We asked respondents whether they agreed with Lyme disease–related statements observed by TERC and TDPL researchers to be common disputed topics, with correct answers dependent on publicly accessible, CDC-promoted information: (1) Lyme disease is unlikely to be transmitted before 24-hours of blacklegged tick attachment (CDC-supported), (2) DEET is safe to use on children (CDC-supported), and (3) antibiotic treatment should be administered regardless of length of tick attachment (not CDC-supported). Finally, the survey contained basic demographic questions and questions to ascertain frequency of social media use. We asked participants to report their resident zip codes and grouped respondents’ states into 9 regions [24], as well as categorized states according to either high-Lyme-disease-incidence (HLI) cases (Connecticut, Delaware, Maine, Maryland, Massachusetts, Minnesota, New Hampshire, New Jersey, New York, Pennsylvania, Rhode Island, Vermont, Virginia, and Wisconsin), or low-Lyme-disease-incidence (LLI) states (all other states) [30].

### Statistical Analysis

We cleaned and analyzed all data using R/RStudio (versions 3.6.1/1.2.5001) [31,32]. We compared demographic variables for TDPL- and TERC-sourced responses using Pearson chi-square test (*stats* package) [31] with simulated *P* value (Monte Carlo simulation based on 2000 replicates) [33] to account for the relatively small sample size of each of these groups. Finding no statistical difference between demographic representation or responses to TDPL- versus TERC-initiated surveys, we aggregated data from both TDPL and TERC surveys. Responses were removed from the final data set if a zip code was not provided because of the inability to examine responses in conjunction with the relative incidence of Lyme disease. However, incomplete surveys that included a zip code location were included in the final analysis, and separate sample sizes were noted in reporting where needed.

We first described the survey population’s reported trust in sources of Lyme disease prevention information, where respondents seek out information first, on what do people base their prevention decisions, and views on the disputed topic statements. To understand whether there were associations among trust in various tick bite prevention sources and recommendations among different demographic data, confidence in prevention ability, and responses to disputed topics, we performed Pearson Chi-square analysis to identify significant univariate correlations and strength of relationships. Finally, we used the *multinom* function from the *nnet* package [34], and the *polr* function within the *MASS* package [34] to fit multinomial and ordinal regression models that determined associations with reported first sought prevention resources, personal prevention confidence, and views on 3 science-based, disputed Lyme disease prevention statements that were determined to be significant in the univariate analysis.

### Ethics Approval

All questions were optional, only those 18 years of age and older were permitted to respond to the survey, and no incentive for participation was provided. The survey and investigation were approved and overseen by the Western Connecticut State University Institutional Review Board (Protocol #1718-19), with an interinstitutional agreement with the University of Rhode Island.

## Results

### Respondent Demographics

Between October 2017 and January 2019, a total of 3480 individuals were reached by the survey advertisement on TERC’s Facebook page (ie, saw the survey but did not necessarily click on it) and 1054 engaged with the survey link (ie, clicked on or shared, but did not begin the survey). On TDPL’s Facebook page, a total of 12,431 people were reached, of which 84 people shared the survey link, and 1499 people were engaged (ie, a total of all interactions including post views, survey link clicks, and post shares). A total of 1190 respondents completed the survey, with 35.6% (424/1190) originating from TDPL sites and 64.3% (766/1190) originating from TERC sources (Table 1). The participants were mostly female (903/1190, 76.3%), nearly half were between 40-59 years of age (574/1182, 48.5%), and a majority reported frequent social media use (850/1188, 71.1% use social media at least once daily; Table 1). Most respondents (995/1190, 83.6%) resided within HLI states [30] (Table 1).

**Table 1:** Survey respondent (N=1190) demographics.

Demographic category and response		Respondents, n (%)
**Survey origin (combined social media platforms; N=1190)**		
	TickEncounter Resource Center	766 (64.3)
	Tickborne Disease Prevention Laboratory	424 (35.6)
**Age group (years; n=1182)**		
	18-29	49 (4.1)
	30-39	183 (15.5)
	40-49	293 (24.8)
	50-59	281 (23.8)
	60-69	269 (22.8)
	70 or older	107 (9)
**Gender (n=1183)**		
	Female	903 (76.3)
	Male	272 (23)
	Nonbinary and other	8 (0.7)
**Region^a^ (n=1190)**		
	New England: Maine, Vermont, New Hampshire, Massachusetts, Rhode Island, and Connecticut	663 (55.7)
	Middle Atlantic: New York, New Jersey, and Pennsylvania	261 (21.9)
	South Atlantic: West Virginia, Maryland, Delaware, Virginia, North Carolina, South Carolina, Georgia, Florida, and District of Columbia	101 (8.5)
	East North Central: Wisconsin, Michigan, Illinois, Indiana, and Ohio	65 (5.5)
	West North Central: North Dakota, South Dakota, Minnesota, Iowa, Nebraska, Kansas, and Missouri	31 (2.6)
	West South Central: Texas, Oklahoma, Louisiana, and Arkansas	24 (2)
	Pacific: Washington, Oregon, California, Alaska, and Hawaii	22 (1.8)
	East South Central: Alabama, Kentucky, Mississippi, and Tennessee	13 (1.1)
	Mountain: Montana, Colorado, Wyoming, Utah, Idaho, Nevada, New Mexico, and Arizona	10 (0.8)
**Resident state Lyme disease incidence^b^ (n=1190)**		
	High incidence	995 (83.6)
	Low incidence	195 (16.4)
**Social media usage (n=1188)**		
	Several times daily	657 (55.3)
	Once daily	193 (16.2)
	A few times a week	117 (9.9)
	One or a few times a month	57 (4.8)
	Less than once a month	42 (3.5)
	I never use social media websites	122 (10.3)

^a^Region based on the study of Hook et al [24].

^b^High-Lyme-disease-incidence states include CT, DE, MA, MD, ME, MN, NH, NJ, NY, PA, RI, VA, VT, and WI. Low-Lyme-disease-incidence states are the remaining 36 states [30]. Heavy social media users are defined as those who access a platform several times daily. Respondents who trust traditional experts are defined as those who reported the highest level of trust in prevention information from their personal physicians, the Centers for Disease Control (CDC), and pest control companies (versus friends and family, web-based forums, Lyme-literate medical doctors, or internet-based health websites; eg, WebMD).

### Reported Trust in Tick-Borne Disease Prevention Resources

A total of 76% respondents reported that, in the past year, they sought out information on the internet about ticks or preventing Lyme disease, and predominantly sought and trusted TBD prevention information from conventional public health sources. Over 80% (3928/4731) of answers reflected that respondents were “very likely” or “somewhat likely” to trust traditional expert category sources of TBD prevention information (Table 2), which included the CDC or another government health agency, one’s primary physician, a university scientist or other academic expert, or mainstream internet health websites (eg, WebMD). University scientists and the CDC or other government health agencies had the greatest reported trustworthiness among traditional sources of TBD prevention information (Table 2). Comparatively, a total of 62% (2937/4731) of responses identified that respondents were “very likely” or “somewhat likely” to trust unconventional sources of TBD information (Table 2), which included Lyme-literate physicians, friends and family, web-based forums and social media, and pest control operators. Lyme-literate physicians and web-based forums and social media were the most trusted among the nontraditional options (Table 2). When asked from which resource do respondents first seek TBD prevention information, they reported university scientists and academic tick resources (248/944, 27.7%), the CDC and government public health agencies (174/944, 19.4%), and WebMD or other mainstream internet health websites (147/944, 16.4%) as the top 3 resources (Table 3).

**Table 2:** The percentage and number of respondents who rated their trust in tick-borne disease prevention information from the sources provided.

Expert status and prevention resource		Likelihood to trust resource for tick-borne disease prevention information			
		Very likely	Somewhat likely	Not that likely	Not at all likely
**Traditional scientific tick-borne disease prevention resources, n (%)**					
	CDC^a^ or government health agency (n=1186)	650 (54.8)	329 (27.8)	109 (9.2)	98 (8.2)
	Primary care physician (n=1188)	436 (36.7)	510 (42.9)	171 (14.4)	71 (6.0)
	University scientist (n=1172)	640 (54.6)	459 (39.1)	58 (5)	15 (1.3)
	Internet health websites (n=1185)	226 (19.1)	678 (57.2)	209 (17.6)	72 (6.1)
	Total	3928 (83)	3928 (83)	803 (17)	803 (17)
**Nontraditional and personal network tick-borne disease prevention resources, n (%)**					
	“Lyme-literate” physician (n=1183)	699 (59.1)	420 (35.5)	49 (4.1)	15 (1.3)
	Friends and family (n=1173)	71 (6)	589 (50.2)	432 (36.8)	81 (7)
	Web-based forum and social media (n=1183)	98 (8.3)	614 (51.9)	382 (32.3)	89 (7.5)
	Pest control operator (n=1185)	41 (3.4)	405 (34.1)	557 (47.1)	182 (15.4)
	Total	2937 (62)	2937 (62)	1787 (38)	1787 (38)

^a^CDC: Centers for Disease Control.

**Table 3:** Sources where respondents reported they first seek tick-borne disease prevention information (n=944): Google searches, Lyme advocacy groups (Lyme Disease Association, International Lyme and Associated Diseases Society, etc), veterinarian, nontraditional medical practitioners (herbalists, naturopaths, and acupuncturists), Cooperative Extension, and friends who are scientists and entomologists.

First-sought source of prevention information	Respondents, n (%)
University scientists and academic tick resources	248 (27.7)
CDC^a^ or other government public health agency	174 (19.4)
WebMD or other mainstream internet health website	147 (16.4)
Personal physician	95 (10.6)
Web-based forum or social media group	72 (8)
Friend, neighbor, or family member	56 (6.2)
Entomology website	33 (3.7)
Google search	27 (3)
Lyme disease advocacy groups	25 (2.8)
Homeopathic practitioners and resources	10 (1.1)
Veterinarian	10 (1.1)

^a^CDC: Centers for Disease Control.

### Factors Associated With Trust in Tick-Borne Disease Prevention Resources

**Figure 1:**
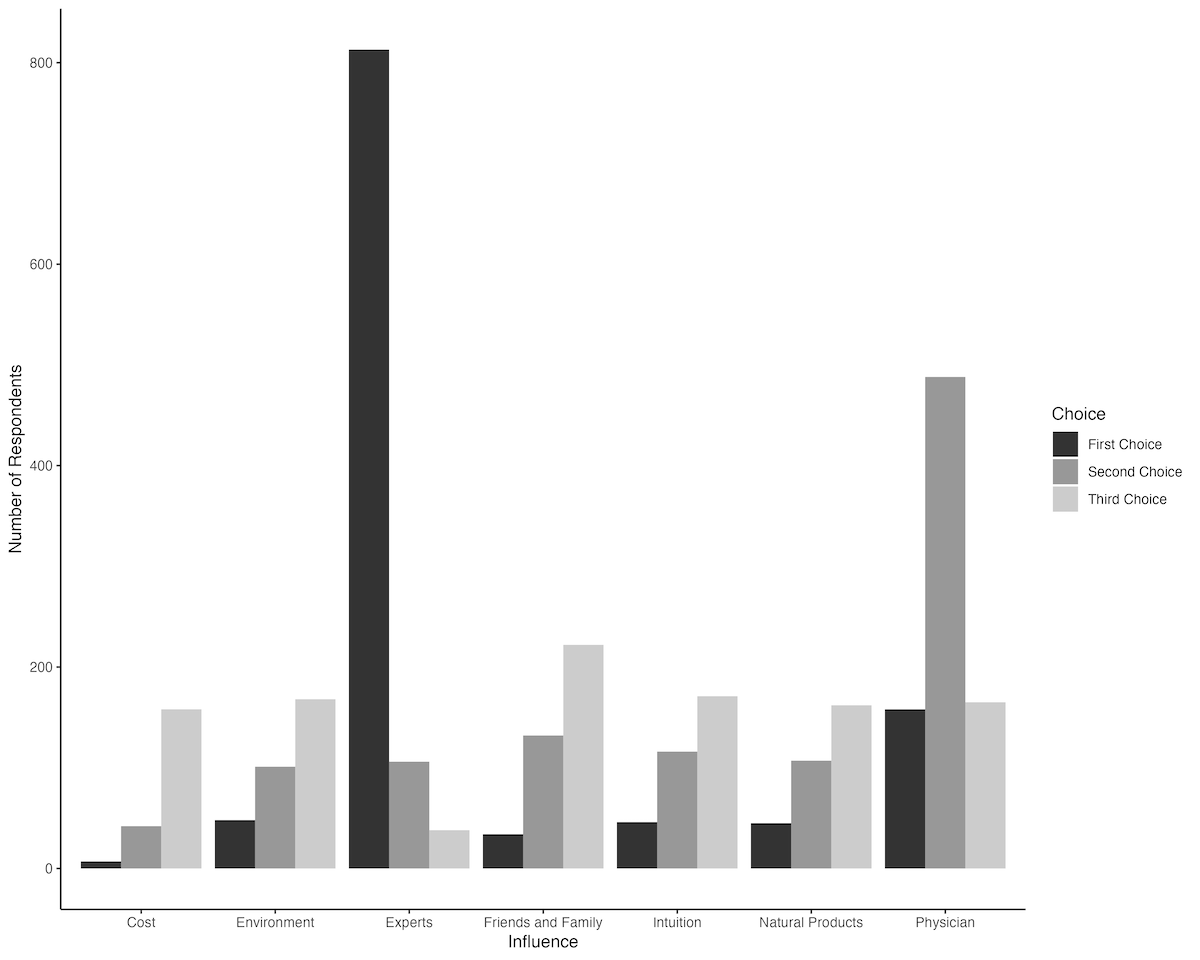
Tick-borne disease prevention product decisions ranked by 7 influential factors (product cost, concern for the potential environmental or health impacts of the product, guidance from public health experts, opinions of friends and family, a desire to use natural or organic products, and guidance of personal physician; N=1151).

Participants from both LLI and HLI states were more likely to first seek TBD prevention information from personal networks and nontraditional sources before traditional sources of TBD prevention information, but those from HLI states were far more likely to first seek out information from personal networks and nontraditional resources (*χ*^2^_3_=146.1, *P*<.001; odds ratio [OR] 0.12, CI –0.57 to 0.082) (Table 4). Self-reported heavy social media usage (defined as frequenting a platform one or more times per day) was associated with an increased likelihood of first seeking a traditional source of prevention information (*χ*^2^_5_=1571.9, *P*<.001; OR 2.25, CI 1.76 to 2.75; Table 4). Further, those who disagreed with CDC-based prevention statements were also more likely to first seek out traditional expert information sources of prevention information (Table 4). Among major factors in tick bite prevention decisions, survey participants revealed the most important influences to be “public health experts” (813/1151, 70.6% of first choice of prevention information), followed by “personal physicians,” (488/1092, 44.6% of second choice of prevention information), and “friends and family” (222/1092, 20.3% of third choice prevention information; Figure 1). These 3 choices comprised the largest proportion of each ranking category. Product cost and concern for the environmental impact of prevention decisions comprised roughly 12.8% (162/1264) and 12.5% (158/1264) of third choice decision criteria (Figure 1).

**Table 4:** Odds ratio (OR) and CI from fitted multinomial regression models for significant variables associated with first seeking traditional expert sources for tick-borne disease prevention information. Traditional expert resources included the Centers for Disease Control (CDC) or another government health agency, one’s primary physician, a university scientist or other academic expert, or mainstream internet health websites (eg, WebMD). Heavy social media usage is defined as 1 or more visits to a platform per day.

	Response: first seeks traditional expert sources of prevention information, OR (CI)
Resides in low-Lyme-disease-incidence state	0.160^a^ (–0.671 to 0.990)
Resides in high-Lyme-disease-incidence state	0.123^a^ (–0.572 to 0.819)
Heavy social media use frequency	2.256^a^ (1.762 to 2.749)
Disagrees that Lyme disease is unlikely transmitted if tick is attached for less than 24 hours	1.986^a^ (1.538 to 2.433)
Disagrees that DEET^b^ is safe for use on children	1.839^a^ (1.400 to 2.277)
Agrees that one should always ask for a full course of antibiotics from physician after any tick bite	1.715^c^ (1.291 to 2.139)
Uncertain if one should always ask for a full course of antibiotics from physician after any tick bite	0.512^c^ (–0.074 to 1.099)
Akaike Information Criterion	704.282

^a^*P*<.01.

^b^DEET: diethyltoluamide.

^c^*P*<.05.

### Confidence in Personal Tick-Borne Disease Prevention Capacity and Associated Factors

**Figure 2:**
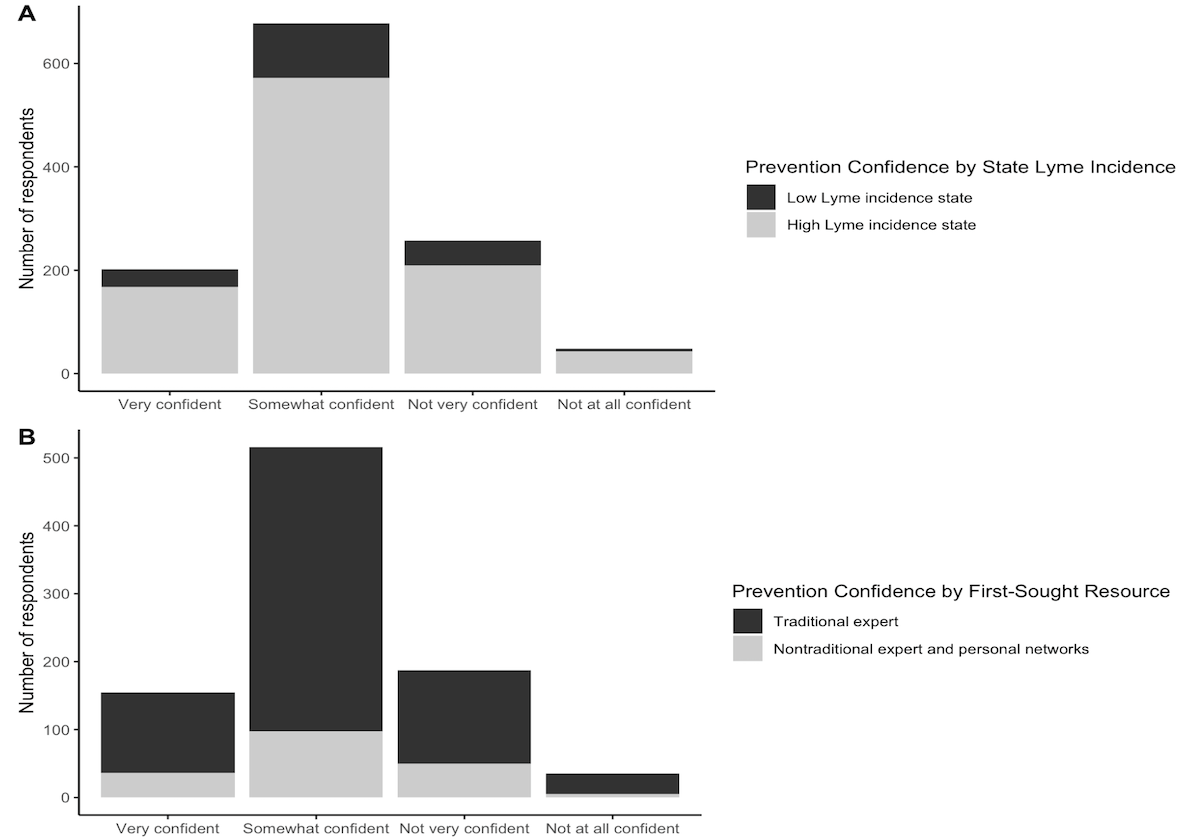
(A) Self-reported confidence in personal tick bite prevention ability by state Lyme incidence (χ2=225.1; *P*<.001; n=1190). High-Lyme-disease-incidence states (n=995) included CT, DE, MA, MD, ME, MN, NH, NJ, NY, PA, RI, VA, VT, and WI [28]. Low-Lyme-disease-incidence states (n=195) included respondents from all other states. (B) Self-reported confidence in personal tick bite prevention ability by first sought prevention resource category (χ2=594.8; *P*<.001; n=1185). Traditional experts (n=707) included personal physician, WebMD or other Internet health website, entomology website, Centers for Disease Control (CDC) or other government public health agency, university scientists and academic tick resources, and veterinarians. Nontraditional experts and personal networks (n=190) included web-based forum or social media group, friend, neighbor, or family member, Google search, Lyme disease advocacy organizations, and homeopathic practitioners and resources.

Most respondents reported relatively high confidence in their ability to protect themselves with 17% of them being “very confident” and 57.2% being “somewhat confident” (Figure 2A and B). Slightly more than one quarter of respondents were either “not very confident” or “not at all confident” in their ability to protect themselves against TBD (Figure 2A and B). Respondents living in LLI states revealed significantly greater confidence in personal TBD prevention ability than those living in HLI states (*χ*^2^_3_=594.8; *P*<.001; Figure 2B), however resident state Lyme disease incidence did not significantly contribute to an ordinal regression model predicting factors involved with prevention confidence (*P*=.31). Respondents’ personal prevention confidence was negatively associated with their social media usage frequency. In general, those reporting less frequent social media usage expressed greater confidence in their ability to protect themselves from tick bites (*χ*^2^_15_=32.2; *P*=.006), but this also was not a significant factor when fitting ordinal regression models. Ordinal regression models predicted that participants who disagreed with the conventional scientific view on the antibiotic prophylaxis prevention statement were less likely to be confident in their ability to protect themselves from a tick bite (OR 0.644, CI 0.309-0.980; *P*=.005). Overall, uncertainty in one’s ability to protect against tick bites was strongly associated with uncertainty about beliefs in CDC-promoted TBD prevention information.

### Participant Agreement With Disputed Lyme Disease Prevention Topic Statements

Respondents were largely uncertain about or in disagreement with CDC-promoted statements about Lyme disease and tick bite prevention. Most disagreed (522/1190, 43.9%) or were uncertain (186/1190, 15.6%) whether Lyme disease is unlikely to be transmitted if a tick is removed within 24 hours of attachment (Table 5). More than half of the respondents disagreed (338/1183, 28.6%) or were uncertain (226/1183, 22.5%) about whether repellent containing DEET is safe to use on children (Table 5). However, nearly half (46.6%) of the respondents felt that it was not necessary to ask their physician for a full course of antibiotics regardless of the length of tick attachment (Table 5).

Acceptance of TBD prevention statements differed by reported trust in expert sources of prevention information. Among those who first sought traditional academic experts for prevention information, nearly half disagreed with CDC-promoted Lyme disease prevention statements (Table 5). Acceptance of science-based information about Lyme disease transmission, DEET safety, and appropriate antibiotic prophylaxis also differed across respondent demographics, specifically state Lyme incidence and reported social media usage frequency. Respondents living in HLI and LLI states represented significantly different beliefs regarding time to transmission of Lyme disease (*χ*^2^_2_=15.5; *P*<.001), and whether one should ask a physician for a full course of antibiotics following a tick bite (*χ*^2^_2_=12.2; *P*=.02; Table 5). Respondents from LLI states were strongly associated with uncertainty about the time-to-transmission statement, while responses from HLI states were more likely to agree that ticks attached for less than 24 hours were unlikely to transmit Lyme disease (OR 0.478, CI –0.176 to –1.133; *P*=.004; Table 6). When considering prophylactic antibiotic administration for any tick bite, HLI state participants were more likely to be uncertain on science-based guidance (OR 0.549, CI –0.036 to 1.135; *P*=.003; Table 6). Respondents who disagreed that DEET products are safe to use on children when applied according to instructions were more likely to trust nontraditional sources of TBD prevention information (OR 2.177, CI 0.891 to 1.899; *P*=.01; Table 6). Those who reported heavy social media usage were more likely to report views inconsistent with CDC public health information than those who used social media less frequently, specifically disagreeing with information on the time to transmission of Lyme disease statement (*χ*^2^_10_=30.4; *P*<.001; OR 1.86, CI 1.259 to 2.464; Table 6).

**Table 5:** Percentage responses to disputed Lyme disease and tick bite prevention statements by 5 categories of respondents to the survey. High-Lyme-disease-incidence states represent responses from people residing within the 14 states identified by the Centers for Disease Control (CDC) to report 95% of all human cases [30]. Heavy social media users are defined as those who reported at least once daily usage of a platform. Traditional experts were defined as CDC or another government health agency, one’s primary physician, a university scientist or other academic expert, or mainstream internet health sites (eg, WebMD). Statistical comparison is among answer choices within each question.

Answer choices			All respondents^a^		Heavy social media users^b^		Those who first seek traditional experts^a^		High-Lyme-disease-incidence states		Low-Lyme-disease-incidence states
“**If a deer tick is attached for less than 24 hours it is unlikely to transmit Lyme disease-causing bacteria to humans.” n (%)**											
	Agree (CDC-supported)	482 (40.5)		*313*^a^ (36.8)		230 (32.7)		*425*^a^ (42.7)		57^a^ (29.2)	
	Disagree	522 (43.9)		*398*^a^ (46.8)		345 (49.1)		*428*^a^ (43)		94^a^ (48.2)	
	Uncertain	186 (15.6)		*139*^a^ (16.4)		128 (18.2)		*142*^a^ (14.3)		44^a^ (22.5)	
“**Skin repellents that contain the chemical DEET are safe to use on children when used according to product directions.” (n=1183), n (%)**											
	Agree (CDC-supported)	579 (48.9)		*397 (47.0)*		371 (52.7)		*473 (47.5)*		*106 (54.6)*	
	Disagree	338 (28.6)		*256 (30.3)*		179 (25.5)		*290 (29.1)*		*48 (24.8)*	
	Uncertain	226 (22.5)		*192 (22.7)*		153 (21.8)		*226 (22.7)*		*40 (20.6)*	
“**You should always ask your doctor for a full course of antibiotics after receiving a tick bite even if you do not know how long the tick was attached or if the tick was infected with Lyme bacteria.” (n=1181), n (%)**											
	Agree	408 (34.5)		310^a^ (36.7)		230 (32.7)		290^c^ (29.4)		58^c^ (30)	
	Disagree (CDC-supported)	550 (46.6)		381^a^ (45.1)		345 (49.1)		390^c^ (39.6)		54^c^ (27.8)	
	Uncertain	223 (18.9)		153^a^ (18.1)		128 (18.2)		306^c^ (31.0)		82^c^ (42.2)	

^a^*P*<.001.

^b^“Heavy social media users” and both high- and low-state Lyme disease incidence categories had different response rates for each question. The denominators for each disputed question among this group are identified and the corresponding values are given in italics.

^c^*P*<.01.

**Table 6:** Odds ratio (OR) and CI from fitted multinomial regression models for significant variables associated with responses to 3 disputed Lyme disease prevention topics. Traditional expert resources included the Centers for Disease Control (CDC) or another government health agency, one’s primary physician, a university scientist or other academic expert, or mainstream internet health websites (eg, WebMD). Heavy social media usage is defined as 1 or more visits to a platform per day and infrequent use is defined as less than once per day usage of a social media platform. Statistically significant associations are denoted with superscripted letters.

Answer choices			Response		
			Disagree, OR (CI)		Uncertain, OR (CI)
**(A) “If a deer tick is attached for less than 24 hours it is unlikely to transmit Lyme disease-causing bacteria to humans.”**					
	Infrequent social media use	1.243 (0.618 to 1.867)		0.186^a^ (–0.840 to 1.213)	
	Heavy social media use frequency	1.756^b^ (1.165 to 2.347)		0.244 (–0.743 to 1.230)	
	Resides in high-Lyme-disease-incidence state	0.523^c^ (0.029 to 1.017)		0.482 (–0.169 to 1.133)	
	First seeks nontraditional expert and personal networks for prevention information	2.554^a^ (2.137 to 2.971)		1.302 (0.677 to 1.926)	
	Not at all confident to prevent tick bites	2.065 (1.081 to 3.050)		8.272^a^ (6.957 to 9.588)	
	Not very confident to prevent tick bites	1.047 (0.523 to 1.570)		3.699^a^ (2.779 to 4.619)	
	Somewhat confident to prevent tick bites	0.986 (0.557 to 1.415)		2.796^c^ (1.953 to 3.639)	
	Akaike Information Criterion	1385.580		1385.580	
**(B) “Skin repellents that contain the chemical DEET are safe to use on children when used according to product directions.”**					
	Infrequent social media use	0.176^a^ (–0.532 to 0.885)		0.126^a^ (–0.664 to 0.916)	
	Heavy social media use frequency	0.224^a^ (–0.438 to 0.886)		0.193^a^ (–0.544 to 0.929)	
	Resides in high-Lyme-disease-incidence state	1.395 (0.891 to 1.899)		2.032^c^ (1.426 to 2.638)	
	First seeks nontraditional expert and personal networks for prevention information	2.177^a^ (1.758 to 2.596)		1.218 (0.736 to 1.700)	
	Not at all confident to prevent tick bites	3.681^a^ (2.717 to 4.644)		2.039 (0.964 to 3.114)	
	Not very confident to prevent tick bites	1.771^b^ (1.165 to 2.378)		2.052^c^ (1.451 to 2.653)	
	Somewhat confident to prevent tick bites	1.844^c^ (1.335 to 2.352)		1.242 (0.716 to 1.768)	
	Akaike Information Criterion	1450.477		1450.477	
**(C) “You should always ask your doctor for a full course of antibiotics after receiving a tick bite even if you do not know how long the tick was attached or if the tick was infected with Lyme bacteria.”**					
	Infrequent social media use	1.154 (0.512 to 1.796)		0.626 (–0.183 to 1.435)	
	Heavy social media use frequency	0.824 (0.221 to 1.427)		0.494^b^ (–0.262 to 1.250)	
	Resides in high-Lyme-disease-incidence state	0.874 (0.382 to 1.366)		0.549^c^ (–0.036 to 1.135)	
	First seeks nontraditional expert and personal networks for prevention information	0.473^a^ (0.072 to 0.874)		0.424^a^ (–0.144 to 0.991)	
	Not at all confident to prevent tick bites	1.345 (0.460 to 2.231)		1.169 (–0.084 to 2.423)	
	Not very confident to prevent tick bites	2.118^a^ (1.580 to 2.656)		2.056^b^ (1.331 to 2.781)	
	Somewhat confident to prevent tick bites	2.039^a^ (1.591 to 2.487)		1.938^c^ (1.325 to 2.551)	
	Akaike Information Criterion	1430.058		1430.058	

^a^*P*<.01.

^b^*P*<.10.

^c^*P*<.05.

## Discussion

### Principal Findings

We used an internet-based survey among people connected to academic-based TBD prevention resources to identify trusted sources of TBD prevention information, which sources are sought out first, how confident respondents are in their ability to protect themselves from TBDs, and how reported trust and confidence are associated with views on disputed TBD prevention topics. Our prediction that those who reported greater trust in established traditional expert public health sources (eg, the CDC and university scientists) would also be more likely to accept CDC-based recommendations for TBD prevention, and report greater confidence in the ability to prevent a tick bite was not entirely supported, and we found that relationships among these variables are often much more complicated.

### Trust

Over 80% (984/1186) of respondents reported high likelihood of trust in conventional sources of TBD prevention information with the CDC and university scientists reflecting the greatest amount of trust. However, our investigation revealed several associations among academic resource–connected internet users living in predominately HLI areas. It appears that while this group seeks an accurate resource for effective prevention science, and reportedly trusts academics to provide TBD information, concurrent distrust in many reputable sources remains. While there is evidence of engaging with academic resources based on acceptance of evidence-based prevention statements, this group also revealed reliance on the opinions of family members, those participating in internet forums, or internet health websites. This result is akin to findings from a previous study that found a majority (62.4%) of survey respondents reported high trust in their physicians despite first seeking medical information on the internet before consulting a medical professional [35]. Those who disagreed with CDC-promoted prevention information on 3 designated disputed topics were the most untrusting of traditional government and academic sources of tick prevention information. Those respondents residing in HLI areas who reflect distrustful attitudes toward evidence-based prevention information are therefore at potentially higher risk for acquiring a TBD. However, the unique position of this group through their voluntary consumption of academic-resource information provides opportunity for building trust and empowering decision-making by increasing targeted education but it requires work to understand and identify reasons for their distrust.

### Confidence

This study firmly documents that the uncertainty expressed in one’s ability to protect oneself against ticks is strongly correlated with uncertainty about beliefs in established TBD prevention information. Respondents engaged with an established academic prevention resource were generally no different in their reported acceptance of CDC-promoted prevention statements except for belief surrounding the time to transmission of Lyme disease, whereas those who were less engaged were uncertain, and that more engaged reflected CDC guidance. Evidence for the ability to establish an engaged audience stems from each group reporting that they first go to the respective organization for prevention information. Those from LLI states reported strong uncertainty related to established TBD prevention information, while those from HLI states were associated with accepting CDC-promoted prevention information for all statements but both groups expressed common concern regarding pesticides [17,18]. Reliance on personal relationships and networks appears paramount, and these support groups can also be used to disseminate practical and evidence-based information [36]. Overall, increased reported social media usage is associated with distrust in mainstream information related to prevention of TBDs.

Our prediction that those who report trust in traditional expert sources were more likely to accept CDC-promoted statements for TBD prevention was supported. However, we found that despite first seeking out, and reportedly trusting scientifically established TBD prevention information, most respondents still held views that are not supported in the scientific literature. This suggests that there are still communication failures among scientific resources of TBD prevention information, and misperceptions are not being addressed in a way that is effectively persuasive. Science communication research has established that providing either the “correct” information or additional clarification of information to the public does not necessarily change misperceptions. The information deficit model of science communication [37] asserts that people are misinformed simply because they lack knowledge, and that scientists can deliver that knowledge in a 1-way direction to solve the knowledge “deficit.” Numerous studies have disproven this hypothesis and demonstrated that approaching science communication without a contextual understanding of the knowledge through a rhetorical lens not only fails to persuade the public [38,39] but erodes trust in the scientific community [40]. Further, scientists risk triggering a “backfire effect” that results in more steadfast adherence to, and cognitive protection of, the misperception when attempting to educate the public without understanding the specific concerns around disputed issues, such as pesticide usage and Lyme disease transmission [41]. Though some researchers have found this phenomenon to be elusive [42], consideration to avoid deficit model communication is prudent.

There are several limitations to this study. The survey was disseminated through social media and web pages specifically to those already connected to academic sources of TBD information. Respondents from this targeted group reside heavily in the Northeastern United States, so these results cannot be generalized to a broader geographic audience. We used the term “Lyme-literate” physician in the survey because it is a commonly recognized practitioner term within this study sample, but it is possible that some of the survey participants were unfamiliar with it or interpreted this term differently than intended by the study authors. Further, in this study, we did not assess why respondents held the views that they expressed, or why they trust certain resources over others. The researchers did not ascertain whether survey respondents have a history of TBDs, or measure respondents’ perception of their own risk. These data are important and should be examined in future works due to the link between one’s history of experience with a TBD and how risk perception can influence tick bite prevention behaviors and beliefs [23,43]. Nonetheless, it is likely that the survey participants perceive they are at risk, given that they chose to engage with social media and internet sources that focus on TBD prevention. This observation is also consistent with research that demonstrated that those identifying as women (ie, most of the respondents) are associated with increased susceptibility and fear regarding Lyme disease compared to men [44], and that those who are diagnosed with chronic Lyme disease tend to be female [45]. When asking about social media usage, we did not identify specific platforms that could affect the type of information and resources that a person relies upon. For those who said they trusted personal relationships or networks, we cannot discern the content of those networks and whether they included scientists or were comprised primarily of lay people. As information within these areas changes over the years, perceptions expressed by study participants can also be expected to shift. This study’s results for this period nonetheless indicate that significant communication barriers exist between public and scientific prevention resources, and these barriers may prevent people from being protected from tick-borne illness or fail to dissuade fear regarding whether they have been infected with a TBD.

While a paradigm shift toward effective mainstream science communication is slow [46], our findings indicate a need to improve social media and internet-based sources of TBD prevention information. We recommend that TBD researchers take small, practical steps to improve public trust in evidence-based information. Meeting the public where they spend time and engaging them on social media platforms is critically important, but mere engagement with the public is not enough to persuade them to use specific prevention methods or gain trust regarding established public health information [40,47]. Attempts to engage in a 2-way dialogue often fail because scientists fall back into comfortable patterns of deficit model communication [48]. Instead, scientists must rely on theoretical communication science when planning internet engagement to increase adoption of prevention behaviors and approach these encounters with empathy and willingness to listen to why the public has come to certain conclusions [49,50]. An important first step is to reframe scientists’ view of “the public” from an othered, nonscientific subclass to one in which they are active participants in the scientific process and discussion [46]. One potentially useful and research-vetted strategy is conducting community-based research to prioritize topics, projects, and discussion of concern that is relevant to the community, in this case time to transmission of Lyme disease, use of pesticides, tick repellents, and antibiotic usage [51]. Using creative communication efforts such as narrative, art, and music in influencing the dissemination and misrepresentation of health information is also a helpful engagement tool and can be used to counter pseudoscience [52]. Ultimately, there is a strong need for studies capable of determining the factors that affect the formation and adoption of beliefs about public health interventions [9], as well as a need for scientists to be open to the study of science communication through resources provided by institutions such as the Alan Alda Center for Communicating Science, and the American Association for the Advancement of Science’s Center for Public Engagement with Science and Technology [46]. Using such strategies where the public engages on social media may improve trust in accurate TBD prevention information and adoption of best disease prevention practices.

## Appendix

Multimedia Appendix 1 These are the 17 questions provided in the survey tool.

## Abbreviations

CDC: Centers for Disease Control
DEET: diethyltoluamide
HLI: high-Lyme-disease-incidence
IDSA: Infectious Disease Society of America
ILADS: International Lyme and Associated Diseases Society
LLI: low-Lyme-disease-incidence
OR: odds ratio
TBD: tick-borne disease
TDPL: Tickborne Disease Prevention Laboratory
TERC: TickEncounter Resource Center

## Data Availability

The data sets generated and analyzed during this study are available from the corresponding author on reasonable request.
