# Supplementary material for: Identifying Trusted Sources of Lyme Disease Prevention Information Among Internet Users Connected to Academic Public Health Resources: Internet-Based Survey Study
Source: JMIR Form Res. 2023 Jul 26;7:e43516. doi: 10.2196/43516 (PMC10413241; doi:10.2196/43516)
Supplement: Multimedia Appendix 1 [file formative_v7i1e43516_app1.docx]

**Supplementary Information – Survey Questions**

**1. State whether or not you agree with each statement (Agree, Disagree, Uncertain):**

1a. If a deer tick is attached for less than 24 hours it is unlikely to transmit Lyme disease-causing bacteria to humans.

1b. Skin repellents that contain the chemical DEET are safe to use on children when used according to product directions.

1c. You should always ask your doctor for a full course of antibiotics after receiving a tick bite even if you do not know how long the tick was attached or if the tick was infected with Lyme bacteria.

1. **Rate how likely you are to trust Lyme disease prevention information that comes from the following sources? (**Very likely, Somewhat likely, Not that likely, Not at all likely**):**

Personal physician

Close friend or family

Online community forum

WebMD or other Internet health site

CDC or other government public health agency

Acquaintance or a friend of a friend

Pest control company

1. **In the last year, have you actively sought out information about ticks or preventing Lyme disease?**

Yes, No

1. **Where did you go FIRST to access information about ticks or preventing Lyme disease?**

Personal physician

Web MD or other Internet health website

Entomology website

CDC or other government public health agency

Friend/neighbor/family member

Online forum or social media group

University scientists and academic tick resources

Google search

Lyme disease advocacy organizations

Homeopathic practitioners and resources

Veterinarian

1. **In the last year, how many times have you clicked on a web link that was shared with you via social media or by email about ticks or Lyme disease prevention?**

0, 1, 2, 3-5, 5-10, more than 10

1. **Did you ever share any of the web links that were shared with you?**

Yes, No

1. **Rate your knowledge about Lyme disease treatment and/or diagnosis**

"I know more than most people"

"I know about as much as most people"

"I know less than most people"

"I do not know about Lyme disease treatment and/or diagnosis"

1. **Rate your knowledge about Lyme disease prevention**

"I know more than most people"

"I know about as much as most people"

"I know less than most people"

"I do not know about Lyme disease prevention"

1. **How often do you use social media websites (e.g., Facebook, Instagram, Twitter, Snapchat, etc.)**

Several times daily

Once daily

A few times a week

Once or a few times per month

Less than once per month

I never use social media websites

1. **Choose the TOP THREE things on which you would base your Lyme prevention decisions (1=most likely, 3=least likely.  You may only use each ranking once)**

Public health experts

Personal physician

Environment

Natural or organic products

Cost

Opinions of friends/family

Intuition

1. **Rate how likely you would be to click on a web link that aims to inform you about the following tick bite prevention measures (Very likely, Somewhat likely, Not that likely, Not at all likely):**

Performing bodily tick checks

Removing a tick from your body or pet

Choosing organic/all-natural tick repellents or yard sprays

Choosing synthetic (non-organic/non-natural) tick repellents or yard sprays

Targeting ticks on rodents in your yard

Diagnosing or treating Lyme disease

Finding a "Lyme-literate" doctor

Identifying a tick

Wearing permethrin-treated tick-repellent clothing

1. **How confident do you feel in your ability to protect yourself and your family from tick bites?**

Very confident

Somewhat confident

Not that confident

Not at all confident

1. **What is your gender?**

Female

Male

Non-binary/Other

1. **What is your age group?**

18-29

30-39

40-49

50-59

60-69

70 or older

1. **Kindly enter your zip code so we can know where our survey data comes from.**
